# Confusion in the Study of Immune Reconstitution Inflammatory Syndrome

**DOI:** 10.20411/pai.v2i1.195

**Published:** 2017-05-02

**Authors:** Claudia Alvarado-De La Barrera, Gustavo Reyes-Terán

**Affiliations:** 1 Departamento de Investigación en Enfermedades Infecciosas, Instituto Nacional de Enfermedades Respiratorias Ismael Cosío Villegas, Mexico City

**Keywords:** HIV, Kaposi's sarcoma, IRIS, Tuberculosis

## Abstract

As a consequence of late presentation for HIV care, a significant proportion of individuals develop immune reconstitution inflammatory syndrome (IRIS) soon after initiation of antiretroviral therapy. Incidence, predictors, and models of pathogenesis of IRIS vary in the literature. Here we discuss factors that may contribute to this lack of consensus. We propose that different pathogens drive different types of IRIS and suggest that these clinical conditions should be studied individually and not grouped under the general heading of “IRIS.”

## INTRODUCTION

In resource-limited settings, a considerable number of individuals with Human Immunodeficiency Virus-1 (HIV) infection develop immune reconstitution inflammatory syndrome (IRIS) as a consequence of late presentation for HIV care. IRIS is an adverse deterioration of clinical status that may occur following successful antiretroviral therapy (ART) [1]. Proportions of such cases range from less than 10% to greater than 50% among those presenting with opportunistic infections [2]. This variation may be caused by differences in underlying pathogens, case definitions used, the study design, the degree of immune compromise, the complexity of clinical presentations, and other factors. IRIS predictors are also variable, but the most consistently reported are a low baseline CD4+ T-cell count [3]; an elevated pathogen load [4]; a short interval between treatment of infection and ART initiation [5]; and high pre-ART and lower on-treatment HIV loads [6].

General case definitions for IRIS have been proposed [7, 8] and the treatment for severe IRIS often includes administration of systemic steroids, despite recognition that effector mechanisms may differ according to the underlying pathogen. Here we revisit the concept that different types of IRIS are determined by the underlying pathogen, and we suggest that these clinical conditions should be studied individually and not grouped under the general concept of “IRIS.” In order to illustrate the differences among these types of IRIS, in which corticosteroids may have profoundly different effects, we reviewed the current literature on tuberculosis-associated IRIS (TB-IRIS) and Kaposi's sarcoma-associated IRIS (KS-IRIS) in the context of HIV infection.

## MYCOBACTERIUM TUBERCULOSIS-ASSOCIATED IRIS

Mycobacterial IRIS is characterized by granulomatous inflammation involving macrophages, epithelioid cells, and multinucleated giant cells surrounded by T lymphocytes [9]. Early findings of an increased number of CD4+ T cells with previously activated or memory phenotype (CD45RO+) after successful ART initiation suggest that MTB-specific T cells have a relevant role in TB-IRIS [10]. Additionally, a peak of PPD specific Th1 cytokines/chemokines [interleukin (IL)-2, IL-12, interferon (IFN)-γ, IFN-γ-induced protein 10 kDa (IP-10), and monokine induced by IFN-γ (MIG)], without Th2 cytokines, and a peak of non-specific inflammatory cytokines/ chemokines (TNF-α, IL-6, IL-1β, IL-10, RANTES, and MCP-1) [11, 12], as well as IL-22 [13], are observed in this condition. TB-IRIS is independently associated with greater increases in IL-6 and TNF-α, IL-8, and G-CSF. Probably the strongest candidate biomarker for predicting TB-IRIS is IL-18 [14].

It is interesting that TB-IRIS is associated with Th1 responses against M. tuberculosis antigens already present before ART and expanded afterward [15]. These preexisting, highly differentiated effector CD4+ T-cells specifically target antigens of the underlying pathogen. That is, individuals with IRIS do not have a generalized T-cell dysfunction. Instead, they have a deregulated CD4+ T-cell response against residual pathogen-derived antigens [16].

As TB-IRIS often occurs before T-cell reconstitution, the role of innate immunity in this condition has received increasing attention [17]. The expression of genes related to pathogen pattern recognition and the complement system is already perturbed before TB-IRIS symptom onset [18]. Circulating CD14++CD16- monocytes, sCD14 and sCD163, are elevated before ART initiation [19]. Despite the peak of Th1 cytokines observed during TB-IRIS events [13], reduced concentrations of granulocyte-macrophage colony-stimulating factor (GM-CSF), G-CSF, IL-3, IL-6, IL-12p40, IL-12p70, IL-15, and IL-17A have been reported before ART initiation in individuals developing TB-IRIS compared to controls with TB and HIV coinfection [20]. As most of these cytokines are mainly produced by monocytes, macrophages, and dendritic cells, this scenario is consistent with the notion that aberrant innate immune responses are partially responsible for the impaired pathogen clearance and elevated antigen loads observed in TB-IRIS.

TB-IRIS is also characterized by aberrant inflammasome activation in monocytes/ macrophages; increased levels of activated caspase1, plasma IL-18, and IL-1β; activation of NLRP3 and AIM2 inflammasomes; and decreased concentrations of the inflammasome activity regulators NO and IFN-γ [21]. Other components of the innate immune response also implicated in TB-IRIS are invariant NKT (iNKT) cells [22]; natural killer (NK) cells CD56+ CD16+ [23]; and neutrophils [24, 25]. The aforementioned mechanisms for TB-IRIS are based on either the adaptive immune response or the innate immune response. However, these mechanisms may be complementary and not mutually exclusive. In fact, an interesting model of IRIS proposes the uncoupling of innate immune system and CD4+ T cells specific for the pathogen-derived antigens [26]. In consequence, we consider that a model of TB-IRIS would involve the essential participation of activated macrophages promoting granulomatous inflammation [9] and producing different non-specific inflammatory cytokines/chemokines, followed by a deregulated specific CD4+ T-cell response against mycobacterial antigens.

Therapeutic approaches for IRIS generally include the specific treatment of the infection, together with continuation of ART. Particularly in TB-IRIS, additional corticosteroid therapy reduced morbidity and improved symptoms and quality of life [27]. The beneficial effects of prednisone in reducing inflammatory reactions are associated with significant decreases in plasma IL-6, IL-10, IL-12 p40, TNF-α, IFN-γ, and IP-10 concentrations, suggesting that steroids may act in TB-IRIS via suppression of proinflammatory cytokine concentrations [28].

## KAPOSI'S SARCOMA-ASSOCIATED IRIS

KS disease progression involves a process of viral oncogenesis within a permissive context of deregulated cytokines and immunocompromise [29]. HIV cooperates at various levels with HHV-8, the etiological agent of KS. HIV Tat directly induces replication [30] and lytic reactivation [31] of HHV-8. HIV Tat also binds to α5β1 and αVβ3 integrins on endothelial and spindle cells, promoting cell adhesion, migration, and cell cycle G1-S progression [32]. In addition, HIV-induced production of TNF-α, INF-γ, IL-1β, and IL-6 induces expression of cell adhesion molecules, matrix metalloproteinase, α5β1 and αVβ3 integrins, endothelial growth factors, and angiogenic cytokines in endothelial cells [33]. Reciprocally, HHV-8 LANA and vFLIP increase HIV replication [34, 35].

Reported incidences for KS-IRIS in HIV-infected populations with KS range from 8.5% in European cohorts and 19.6% in sub-Saharan Africa cohorts [36], to 42.8% in Mexican cohorts [37]. Predictors for KS-IRIS reported in the literature include initial KS treatment with ART alone (hazard ratio, [HR] 2.97); AIDS Clinical Trial Group (ACTG) stage T1 KS disease (HR, 2.96); plasma HIV-1 RNA > 5 log10 copies/ml (HR, 2.14) [36]; clinical KS before ART treatment (HR, 91.4); detectable plasma HHV-8 DNA (HR, 24.4); hematocrit < 30% (HR, 26.45 [38]; and the use of corticosteroids (odds ratio, 2.36) [39].

A mechanism for AIDS-KS pathogenesis based on the abortive lytic and paracrine oncogenesis hypotheses proposes that, in HIV/AIDS, decreased immunosurveillance, inflammatory cytokines, and HIV Tat lead to HHV-8 reactivation and reinfection. Cells expressing oncogenic early lytic genes can be transformed and stimulated in a paracrine fashion by lytically infected cells [40]. The mechanism of KS-IRIS has not been elucidated, but we propose that, based on the aforementioned mechanism for AIDS-KS, IRIS would develop in subjects presenting different qualitative and quantitative combinations of the aforementioned KS-IRIS predictors. In this scenario, the interaction between HIV and HHV-8 would regulate the proliferation, transformation, proangiogenic, and apoptotic cellular pathways. Before ART initiation, elevated levels of HIV Tat would promote HHV-8 reactivation. Another model proposes that, in individuals with a higher HHV-8 dissemination and high HIV load, ART-induced immune reconstitution of HHV-8-specific response may be ineffective in controlling HHV-8 replication, and a cytokine-induced reactive angioproliferation and tumorigenesis might occur, resulting in the development of paradoxical KS-IRIS [36].

Administration of systemic corticosteroids accelerates the clinical progression of KS in HIV-infected individuals [41–46]. We recently reported that the use of corticosteroids is a risk factor for KS-IRIS and KS-associated mortality in the context of HIV infection [39]. Numerous mechanisms could potentially explain the association of steroids with KS disease progression. First, by impairing immune surveillance, corticosteroids may favor oncogenesis. Second, exogenous corticosteroids upregulate steroid receptors, which in turn promotes proliferation of KS spindle cells [47] and HHV-8 activation [48]. Third, corticosteroid stimulation of KS development can also occur through upregulation of oncostatin M and IL-6/sIL-6R [49] and blockade of transforming growth factor-β, an autocrine inhibitory factor for Kaposi's sarcoma [50]. Finally, the association of LANA with corticosteroid receptors promotes HHV-8 lytic reactivation [51].

## DISCUSSION

Dissimilar and contrasting results regarding IRIS incidence, pathogenesis, and risk factors have been reported. There is lack of consensus on IRIS case definitions for each type of IRIS, the identification of useful diagnostic biomarkers and, most importantly, on the therapeutic approach for IRIS. Despite common predisposing factors between different forms of IRIS such as high pathogen burden and low CD4+ T-cell counts, specific IRIS pathways may differ. As examples, KS is a viral-driven tumor, and TB is caused by intracellular bacteria activating macrophages. In these types of IRIS, corticosteroids have contrasting effects: a therapeutic benefit in TB-IRIS [27]; and a deleterious effect in KS-IRIS [39]. Steroids are the blunt immunosuppressives. Based on the pathophysiology of each type of IRIS, one can hope that more elegant and specific therapies will become available. Specific therapies would target key mediators for each type of IRIS. For instance, some potential therapeutic targets for TB-IRIS may include IL-6, TNF-α and IL-18; while potential therapeutic targets for KS-IRIS may include HHV-8 LANA, IL-6, proangiogenic factors, and others. A potential therapeutic target for paradoxical progressive multifocal leukoencephalopathy (PML)-IRIS may be the CCR5 receptor. In a case report, fast improvement after addition of the CCR5 blocker maraviroc to initial ART was observed [52]. However, in a subsequent clinical trial in people with advanced HIV infection, administration of maraviroc added to an initial ART regimen did not confer meaningful protection from the occurrence of IRIS [53]. We might then wonder whether the negative results of this clinical trial could be partially explained by the enrollment of HIV-infected individuals with different types of IRIS. Our concern is that, by grouping HIV-infected individuals with IRIS associated with different pathogens, it is difficult to unravel the complex sequence of events leading to IRIS.

Lack of consensus on the case definitions used has also increased the heterogeneity of the populations studied. Despite specific case definitions proposed for TB-IRIS [54], cryptococcal-IRIS [55], KS-IRIS [56], and cytomegalovirus retinitis-IRIS [57], not all studies have used the same definitions. Discrepancies among IRIS studies may also depend on the samples analyzed. Cell culture supernatants of antigen-stimulated peripheral blood mononuclear cells or serum/plasma samples may not always reflect events occurring in target tissues. In TB meningitis, for example, prednisone significantly reduces cytokines in serum [58] but not in cerebrospinal fluid [59]. Finally, adjunctive corticosteroid treatment for conditions other than IRIS may represent another source of variability, as steroids seem to inhibit many immune elements.

When HIV-infected patients with IRIS associated with different pathogens are analyzed together, there is a risk of incorrectly extrapolating conclusions from one entity to another. An example of this is the inappropriate use of steroids in KS-IRIS, as suggested by the positive experience in TB-IRIS.

## CONCLUSIONS

In order to more accurately evaluate the pathogenesis and treatment of IRIS, we suggest that cases be grouped and analyzed according to the associated pathogen. If necessary, adequate sample sizes would be achieved through multicenter collaborative studies. In addition, we encourage the use of consensus IRIS case definitions to allow consistent interpretation across studies. Moreover, the administration of corticosteroids should be acknowledged as a study limitation if the pathogenesis of IRIS is being explored. Finally, if sampling of target tissues can be achieved safely and ethically, this may help clarify aspects of IRIS pathogenesis.
